# Society Meetings

**Published:** 1897-12

**Authors:** 


### Society Meetings.

The New York State Association of Railway Surgeons held its tenth annual meeting at the Academy of Medicine, No. 17 West Forty-third street, Tuesday, November 16, 1897. Dr. J. Frank Valentine, of Brooklyn, presided, and Dr. C. B. Herrick, of Troy, was the secretary. The morning program included papers by Dr. Thomas H. Manley, of New York ; Dr. W. B. Outten, of St. Louis ; Dr. W. J. Herdman, of Ann Arbor, and Dr. L. L. Gilbert, of Pittsburg. Dr. R. H. Ecclesten, of Providence; William J. Kelly, counsel to the Long Island Railroad, and Drs. Charles L.

Dana, Reginald H. Sayre, and Landon Carter Gray and Mr. Clark Bell, of New York, took part in the discussion which followed. Although the association is exclusively of New York, there were many physicians from other states as guests.

Papers were read in the afternoon by Clark Bell, of New York; Dr. C. B. Herrick, of Troy; Dr. C. S. Parkhill, of Hornellsville; Dr. John F. Burns, of Brooklyn; Dr. W. C. Wood, of Gloversville; Dr. A. F. Palmer, of Mechanicsville; Dr. Harvey P. Jack, of Canisteo and Dr. Charles R. Phillips, of Hornellsville.

W. H. Baldwin, Jr., president of the Long Island Railway Company, made an address.

The following officers were elected: President, Dr. Clinton B. Herrick, of Troy; vice-presidents, Dr. Thomas D. Mills, of Middletown, and Dr. William D. Morrow, of Walton; secretary, Dr. George Chaffee, of Brooklyn; treasurer, Dr. Harvey P. Jack, of Canisteo.

---

THE semi-annual meeting of the Medical Society of the County of Chautauqua will be held at the Humphrey House, Jamestown, Tuesday, December 14, 1897, under the presidency of Dr. M.N. Bemus, of Jamestown. The program includes a business session at 11 o'clock a. m., and a scientific session in the afternoon. The following work is outlined for the latter: Acute peritonitis—(1) Etiology and pathology, V. M. Griswold, Fredonia; (2) Diagnosis of, E. A. Scofield, Bemus Point; discussed by J. E. Caneen, Ripley, J. M. Brooks, Jamestown, (3) Medical treatment, Jason Parker, Jamestown; discussed by N. G. Richmond, Fredonia, A. J. Bennett, Busti; (4) Surgical treatment, Wm. M. Bemus, Jamestown; discussed by E. S. Rich, Kennedy; L. C. Green, Panama; (5) Paper, (subject to be announced,) Dr. C. M. Daniels, Buffalo, N. Y. A cordial invitation is extended to the profession of adjoining counties to be present and take part in the discussions. The Hon. A. C. Wade will address the society at the parlors of the Humphrey House in the evening, at 8 o'clock. This will be followed by a banquet. Dr. C. A. Ellis, of Sherman, is the secretary.

---

THE American Electro-therapeutic Association will hold its next annual meeting at Buffalo, Tuesday, Wednesday and Thursday, September 13, 14 and 15, 1898, under the presidency of Dr. Charles R. Dickson, of Toronto. The secretary is Dr. John

Gerin, 68 North street, Auburn, N. Y., to whom communications relating to the meeting should be addressed.

---

THE Buffalo Academy of Medicine held meetings during the month of November as follows :

*Section on Surgery.*—Thursday evening, November 4th, program : Congenital dislocation of the hip, illustrated by lantern slides, by Dr. E. H. Bradford, surgeon to the Children's Hospital, Boston, Mass., assistant professor of orthopedic surgery, Harvard medical school. Discussion by Dr. Bartow and Dr. Weigel, of Rochester. Exhibition of a case by Dr. Chauncey P. Smith.

*Section on Medicine.*—Tuesday evening, November 9th, program : Renal calculus, by Dr. John H. Musser, professor of clinical medicine, University of Pennsylvania. Discussion opened by Drs. Charles G. Stockton and Herman Mynter.

*Section on Pathology.*—Tuesday evening, November 16th, program : Recent researches in the pathology of trichophytosis, illustrated by stereopticon, by Dr. William Thomas Corlett, M. D., L. R. C. P., Lond., professor of dermatology and syphilology, Western Reserve University, Cleveland, O. Multiple idiopathic pigmented sareoma of the skin, presentation of a case, with remarks, by Dr. Grover W. Wende.

*Section on Obstetrics and Gynecology.*—Tuesday evening, November 23d, program : Diagnosis and treatment of carcinoma of the cervix, by Dr. M. A. Crockett. Report of cases and presentation of specimens, by Drs. Earl P. Lothrop and Francis E. Fronczak.

---

THE Southern Surgical and Gynecological Association held its tenth annual meeting at St. Louis, November 9, 10 and 11, 1897, under the presidency of Dr. George Ben Johnston, of Richmond. In his inaugural address Dr. Johnston made reference to the prevalence of yellow fever in the South, expressing regret that several members were detained there on that account. We make reference in another place to Dr. Johnston's annual address. The Hon. Charles Nagel delivered an admirable address of welcome, to which the president gracefully responded.

Dr. Henry H. Mudd, chairman of committee of arrangements, outlined the program of the meeting, both scientific ands ocial, and then the reading of papers began. These will be fully reported in the journals and finally printed in the transactions. This annual volume is edited by the secretary, Dr. William E. B. Davis, of Birmingham, and is one of the most interesting records of society transactions that is published. This year promises to be no exception to the rule, judging from the wealth of material delivered at St. Louis.

On the evening of the first day, Tuesday, Dr. Mudd gave a reception at his residence that was attended by the wealth and fashion of St. Louis, who came to do honor to the visitors. The elegance of costumes in which the women were arrayed, the exquisite music and the delightful supper, all served to round up a most charming evening. The annual banquet was given at the Southern hotel on Wednesday evening, at which Dr. L. S. McMurtry, of Louisville, acted as master of ceremonies. Dr. McMurtry, whose post-prandial talents are well known, was at his best on this occasion and when he introduced Dr. I. N. Love, who made one of the principal speeches at the banquet, it was a climax. Dr. Love is always equal to a good speech, but never made a better one than on this occasion. Dr. Joseph M. Mathews, of Louisville, and Dr. R. M. Cunningham, of Birmingham, gifted also as after-dinner speakers, made notable addresses on this occasion. Next day the association elected the following-named officers: President, Dr. Richard Douglas, Nashville; vice-presidents, Drs. H. H. Mudd, St. Louis, J. A. Goggans, Alabama; secretary, Dr. W. E. B. Davis, Birmingham; treasurer, Dr. A. M. Cartledge, Louisville. Council, Drs. L. M. Tiffany, Baltimore; Geo. Ben Johnston, Richmond; Lewis S. McMurtry, Louisville; George J. Englemann, Boston; Ernest S. Lewis, New Orleans.

The next meeting will be held at Memphis, Tenn., beginning on the second Tuesday of November, 1898.

---

THE New York Academy of Medicine held its anniversary meeting on Thursday evening, November 18, 1897, at No. 19 West Forty-third street. Dr. A. Alexander Smith delivered an address in memory of the late Dr. William T. Lusk, and the anniversary discourse was delivered by Dr. Hermann M. Biggs, on Sanitary Science, the Medical profession and the Public.

---

THE Chicago Pathological Society will hold its regular meeting Friday, December 3, 1897, at which time Brig.-General George M.

Sternberg, of Washington, D. C., Surgeon-General of the United States Army, will deliver a lecture on the Etiology and Pathology of Yellow Fever. Members of the profession are cordially invited and tickets may be obtained either of Dr. George H. Weaver, 535 Washington Boulevard, Chicago, or from the Railway Surgeon.

